# Host life-history traits predict haemosporidian parasite prevalence in tanagers (Aves: Thraupidae)

**DOI:** 10.1017/S0031182022001469

**Published:** 2023-01

**Authors:** Victor Aguiar de Souza Penha, Fabricius Maia Chaves Bicalho Domingos, Alan Fecchio, Jeffrey A. Bell, Jason D. Weckstein, Robert E. Ricklefs, Erika Martins Braga, Patrícia de Abreu Moreira, Letícia Soares, Steven Latta, Graziela Tolesano-Pascoli, Renata Duarte Alquezar, Kleber Del-Claro, Lilian Tonelli Manica

**Affiliations:** 1 Graduate Program in Ecology and Conservation, Federal University of Paraná, Curitiba, Paraná, Brazil; 2 Zoology Department, Federal University of Paraná, Curitiba, Paraná, Brazil; 3 Centro de Investigación Esquel de Montaña y Estepa Patagónica (CIEMEP), CONICET – Universidad Nacional de la Patagonia San Juan Bosco, Esquel, Chubut, Argentina; 4 Department of Biology, University of North Dakota, Grand Forks, USA; 5 Academy of Natural Sciences of Drexel University and Department of Biodiversity, Earth, and Environmental Science, Drexel University, Philadelphia, PA, USA; 6 Department of Biology, University of Missouri–Saint Louis, Saint Louis, MO, USA; 7 Malaria Laboratory, Federal University of Minas Gerais, Belo Horizonte, Minas Gerais, Brazil; 8 Federal University of Ouro Preto, Ouro Preto, Minas Gerais, Brazil; 9 Research Associate, National Aviary, Pittsburgh, PA, USA; 10 Conservation and Field Research, National Aviary, Pittsburgh, PA, USA; 11 Zoology Department, Institute of Biological Sciences, University of Brasilia, Brasilia, Distrito Federal, Brazil; 12 Animal Behavior Laboratory, Graduate Program in Ecology, University of Brasilia, Brasilia, Distrito Federal, Brazil; 13 Behavioral Ecology and Interactions Laboratory, Graduate Program in Ecology and Conservation of Natural Resources, Federal University of Uberlândia, Uberlândia, Minas Gerais, Brazil

**Keywords:** Diet, habitat type, incubation period, *Parahaemoproteus*, *Plasmodium*, temperature

## Abstract

Vector-borne parasites are important ecological drivers influencing life-history evolution in birds by increasing host mortality or susceptibility to new diseases. Therefore, understanding why vulnerability to infection varies within a host clade is a crucial task for conservation biology and for understanding macroecological life-history patterns. Here, we studied the relationship of avian life-history traits and climate on the prevalence of *Plasmodium* and *Parahaemoproteus* parasites. We sampled 3569 individual birds belonging to 53 species of the family Thraupidae. Individuals were captured from 2007 to 2018 at 92 locations. We created 2 phylogenetic generalized least-squares models with *Plasmodium* and *Parahaemoproteus* prevalence as our response variables, and with the following predictor variables: climate PC1, climate PC2, body size, mixed-species flock participation, incubation period, migration, nest height, foraging height, forest cover, and diet. We found that *Parahaemoproteus* and *Plasmodium* prevalence was higher in species inhabiting open habitats. Tanager species with longer incubation periods had higher *Parahaemoproteus* prevalence as well, and we hypothesize that these longer incubation periods overlap with maximum vector abundances, resulting in a higher probability of infection among adult hosts during their incubation period and among chicks. Lastly, we found that *Plasmodium* prevalence was higher in species without migratory behaviour, with mixed-species flock participation, and with an omnivorous or animal-derived diet. We discuss the consequences of higher infection prevalence in relation to life-history traits in tanagers.

## Graphical abstract

**Figure:**
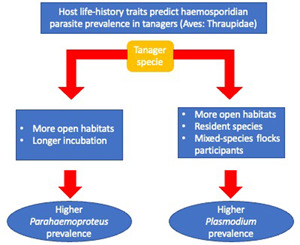

## Introduction

Vector-borne haemosporidian parasites can negatively impact host fitness by mediating life-history trade-offs, such as trading investment in immune defence over investment in plumage coloration in response to infection (Hõrak *et al*., 2001; Delhaye *et al*., 2018; Penha *et al*., 2020). Furthermore, haemosporidian infections have been associated with avian mortality (Permin and Juhl, 2002; Atkinson and Samuel, 2010; Jia *et al*., 2018), and with lower health status in birds (Himmel *et al*., 2021). Haemosporidian parasites cause malaria and related diseases in wild and domesticated birds; these parasites are ecologically and evolutionarily diverse, with a worldwide distribution (Valkiūnas, 2005; Perkins, 2014). Each haemosporidian genus is transmitted to the avian host by a different group of dipteran vectors: *Plasmodium* by mosquitoes (Culicidae) and *Parahaemoproteus* by biting midges (Ceratopogonidae; Santiago-Alarcon *et al*., 2012). Because avian haemosporidian parasites are broadly distributed, common in avian populations, and easily detected in small blood samples, they provide an important and accessible model system for studying host–parasite interactions.

Within an avian community, host exposure to parasites may be influenced by the environment (e.g. climate), and life-history traits of the host species (Svensson-Coelho *et al*., 2014; Canard *et al*., 2015; Lutz *et al*., 2015; Clark and Clegg, 2017). Climate (particularly rainfall and temperature) may play an important role in parasite exposure through its influence on vector development and abundance (Loiseau *et al*., 2011; Gehman *et al*., 2018). For example, in central and west Africa, *Plasmodium* prevalence in the olive sunbird (*Cyanomitra olivacea*) was higher in locations with high temperatures (Sehgal *et al*., 2011). In community level studies, involving several avian host species, temperature also seems to be a good predictor of *Plasmodium* prevalence, such as in northeastern Brazil (Rodrigues *et al*., 2021), and in the Spanish Iberian Peninsula (Illera *et al*., 2017). However, *Parahaemoproteus* prevalence has shown contrasting results (associated with colder environments) in comparison with *Plasmodium* (Clark, 2018; Clark *et al*., 2018, 2020), which may be related to the different life histories of the primary vectors of *Plasmodium* and *Parahaemoproteus* parasites.

Host life-history traits may influence haemosporidian parasite prevalence, since these traits are associated with varying host exposure to vectors (Medeiros *et al*., 2013; Svensson-Coelho *et al*., 2016). Nesting and foraging height, body size, habitat type, flocking, migratory behaviour (Møller and Erritzøe, 1998; Svensson-Coelho *et al*., 2016), and diet (González *et al*., 2014; Turcotte *et al*., 2018; Tchoumbou *et al*., 2020) are all factors that may influence host exposure to vectors (Medeiros *et al*., 2013; González *et al*., 2014; Lutz *et al*., 2015). For example, single- or mixed-species flock participants tend to have a higher haemosporidian parasite prevalence because flocking hosts tend to attract more vectors or simply be in contact with more insects (Fecchio *et al*., 2013; Isaksson *et al*., 2013; Ellis *et al*., 2017), whereas birds foraging and nesting in the canopy and inhabiting closed habitats may have increased parasite prevalence due to a higher vector abundance in these forest strata (Garvin and Greiner, 2003; Swanson and Adler, 2010; Laporta *et al*., 2011; Swanson *et al*., 2012; Ibañez-Justicia and Cianci, 2015; Lutz *et al*., 2015). Host diet may also be an important factor in predicting haemosporidian prevalence, with insectivores harbouring higher prevalence, because of their closer contact with insects, which leads to an increased susceptibility to vectors (Braga *et al*., 2011; González *et al*., 2014). Analyses of the influence of migration on haemosporidian prevalence have shown contrasting patterns; migratory host species have exhibited higher haemosporidian prevalence due to higher pathogen exposure (Ciloglu *et al*., 2020; Anjos *et al*., 2021; de Angeli Dutra *et al*., 2021), but in other studies resident species have exhibited higher haemosporidian prevalence perhaps due to the increased predictability of hosts to vectors through space and time (Slowinski *et al*., 2018; Soares *et al*., 2020). Haemosporidian parasite infection prevalence might also relate to host incubation period (Matthews *et al*., 2016), which is likely associated with avian life-history trade-offs between immune response and the duration of incubation (Ricklefs, 1992). Therefore, birds with longer incubation periods may have an adaptive advantage by having an increased length of time for B-cell maturation, conferring increased protection against infections (Ricklefs *et al*., 2018).

Here, we investigated haemosporidian parasite prevalence in tanagers (Passeriformes: Thraupidae), the largest family of songbirds. Tanager species commonly occur from northern Mexico, through Central America, the Caribbean and South America, accounting for 12% of bird species in the Neotropical region (Parker *et al*., 1996). Tanagers occupy several habitat types, ranging from rainforests to grasslands, with nearly all avian foraging niches being filled by members of the family (Burns *et al*., 2014). Thraupidae currently includes 377 species placed in 15 subfamilies (Burns *et al*., 2016; Winkler *et al.*, 2020). Tanager species have a broad range of complex behaviours, habitat preferences, and morphological characteristics (Macedo *et al*., 2012; Manica and Marini, 2012; Burns *et al*., 2014; Nogueira *et al*., 2014; Lima-Rezende and Caparroz, 2016; Beier *et al*., 2017). Because of this impressive diversity, the accumulated knowledge on tanager ecology (Shultz and Burns, 2017), and the fact that they have been well sampled within the Neotropical region, make them a good model system for studying the effects of host life-history variation and environmental variation on haemosporidian prevalence. Despite recent advances in the study of haemosporidian prevalence of Neotropical birds (Fecchio *et al*., 2011, 2022; Sebaio *et al*., 2012; de Angeli Dutra *et al*., 2021; Ellis *et al*., 2021), there is still a lack of information on the vulnerability of tanager species to haemosporidian parasites. Therefore, in this study we sought to understand the relationships among parasitism by haemosporidians, tanager life-history traits and environmental traits. More specifically, we tested whether haemosporidian parasite prevalence was related to species' nesting and foraging strata, habitat preference in terms of forest cover, participation in mixed-species flocks, diet, migratory behaviour, length of incubation period, environmental temperature regime, and annual precipitation.

## Materials and methods

### Data collection

We assembled haemosporidian screening data from 3569 individual birds belonging to 53 species in the family Thraupidae. Individuals were captured between 2007 and 2018 at 92 locations in 7 countries in the Neotropics, including Argentina (Soares *et al*., 2016; Fecchio *et al*., 2019*a*), Brazil (Lacorte *et al*., 2013; Ferreira *et al*., 2017; Fecchio *et al*., 2019*a*, 2021; Lopes *et al*., 2020; Penha *et al*., 2020; Rodrigues *et al*., 2020), Dominican Republic (Latta and Ricklefs, 2010; Soares *et al*., 2020), Ecuador (Svensson-Coelho *et al*., 2014), Honduras (this study), Mexico (Fecchio *et al*., 2019*b*), and Nicaragua (this study).

### Haemosporidian parasite analysis

To compare lineages identified by our nested PCR protocols to those in the MalAvi database (Bensch *et al*., 2009), we aligned nucleotide sequences using the BIOEDIT v 7.2.0 program (Hall, 1999) and verified sequence identities through a local BLAST against the MalAvi database. MalAvi is a database that groups and standardizes haemosporidian parasite lineages found in various hosts, allowing the study of host–parasite distributions, prevalence, and specializations (Bensch *et al*., 2009). Lineages identified using the protocol that amplified a longer mtDNA fragment (Ricklefs *et al*., 2005; Soares *et al*., 2016, 2020) were successfully matched to known lineages in the MalAvi database only when the 2 fragments had 100% identical nucleotide sequences in their overlapping region (lineage names here are as in the MalAvi database). We calculated the prevalence of each *Parahaemoproteus* and *Plasmodium* lineage separately for every host species as the number of infected individuals divided by the total number of screened individuals (proportion of infected individuals). We treated *Parahaemoproteus* as a distinct genus from *Haemoproteus* (*Haemoproteus*) following recent phylogenetic advancements in the haemosporidian parasite phylogeny (Martinsen *et al*., 2008; Borner *et al*., 2016; Galen *et al*., 2018).

### Host phylogeny

We used the Thraupidae phylogeny from Burns *et al*. (2014), reconstructed with 6 molecular markers, which was the first comprehensive tanager phylogeny; Burns *et al*.'s (2014) phylogenetic hypothesis included genera not found in Jetz *et al*. (2012). This phylogeny produced a highly comprehensive framework for studying macroevolutionary patterns among tanager taxa. We used *ape* (Paradis *et al*., 2004) to prune out species not found in our database from the tree.

### Life-history traits and climate

We used the Handbook of the Birds of the World (Winkler *et al.,*, 2020; https://birdsoftheworld.org/bow/home) to compile the following variables from the 53 tanager species: *body size* (average body length in centimetres); *mixed-flock participation* (participant [frequently or loosely join mixed-species flocks] and non-participant [rarely or does not join mixed-species flocks]); *foraging height* (ground [forages on or close to ground]; understory [forages in the midstory of the forest, understory, shrubs or small trees] and canopy [forages in tall trees, or in the canopy of forests]); *migration status* (migrant or permanent resident – complemented with data from Somenzari *et al*. (2018) for species that occur in Brazil); and *incubation period* (average number of days laying to hatching). We also collected information on *nest height*, including low (0–1 m; on or close to the ground), middle (1–5 m; in shrubs, small trees, understory or mid canopy) and high (>5 m or tall trees and upper canopies). We used the data available in Olson and Owens (2005) to categorize foraging ecology including plant-eating (herbivore: fruits, seeds, leaves and other plant parts), animal-eating (carnivore: arthropods, spiders or others) or a generalist diet (omnivore). We used the data available in the Global Habitat Heterogeneity database (Tuanmu and Jetz, 2015) as a proxy for habitat type (denoted by the variable name ‘*forest cover*’ hereafter). We used occurrence data from eBird (https://ebird.org/data/download) and the *extract* function from the *raster* package (Hijmans, 2021), and then averaged the GHH) (Global Habitat Heterogeneity) values for each species across its distribution. Higher GHH indicates more forested habitats, whereas a lower GHH indicates open habitats. Lastly, we extracted all 19 climatic variables for the capture sites of all individuals (our 92 different capture sites) from WorldClim 2 (Fick and Hijmans, 2017). For each host species, we averaged climatic values from all sites where a given species was captured. Since we could not determine age and sex of all individuals from every species, we did not include these 2 variables in our models. We then performed a principal components analysis to reduce the dimensionality of the climatic variables (summary statistics can be found in Supplementary Table 2 and Fig. 2). The first and second components together explained 68.6% of the variation and were used as our climatic variables (hereinafter climate PC1 and PC2). PC1 was primarily related to temperature and was positively associated with variables such as mean annual temperature, minimum temperature of coldest month, mean temperature of driest quarter and mean temperature of the coldest quarter, whereas PC2 was negatively associated with precipitation variables, such as annual precipitation, precipitation of the driest month, precipitation of the driest quarter, and positively associated with precipitation seasonality.

### Statistical analysis

Using the host phylogeny, we created 2 different phylogenetic generalized least-squares models to test the hypothesis that parasite prevalence is predicted by host-related parameters and climate. For each model we used parasite prevalence (proportion of infected individuals) as the response variable, one for *Parahaemoproteus*, and another for *Plasmodium*. We only considered species with 5 or more captured individuals for analysis with these models (see Supplementary Tables 3–5 and Fig. 3 for a more conservative analysis including species with 10 or more captured individuals). We used the following explanatory variables: climate PC1, climate PC2, body size, mixed-species flock participation, incubation period, migration, nest height, foraging height, forest cover and diet. All numerical variables were standardized using the scale function from R, to remove unwanted variation in the scale among variables. Before including all variables, we tested for multicollinearity using the variance inflation factor (VIF) calculated by using the *VIF* function from the *regclass* package (James *et al*., 2014; Petrie, 2020). We used a conservative threshold of 2 for the values of GVIF^(1/2*df*)^ as a sign of multicollinearity. We found no collinear predictors based on this approach, and therefore all variables were included in the analysis. We tested model convergence with the Ornstein–Uhlenbeck (OU) and Brownian motion (BM) evolutionary models using Akaike information criterion (AIC) values. We then selected the best models using an information-theoretic approach (Burnham and Anderson, 2002) with the *dredge* function in the *MuMIn* package (Barton, 2019). When *w_i_* (weight) of the best model was below 0.80, we used model averaging in the *model.avg* function in the *MuMIn* package to calculate the model-averaged estimates, following the protocol described by Burnham *et al*. (2011). We used root mean square error (RMSE) to validate each model, considering RMSE closest to zero as models with a good fit (Norberg *et al*., 2019; Tobler *et al*., 2019; Snell Taylor *et al*., 2021). We assessed the importance of the explanatory variables by evaluating their estimates, unconditional standard errors and 95% confidence intervals (CIs) in the averaged model. Since foraging, nest height and diet have 3 different levels, we used the *relevel* function to change the reference level of each categorical variable to rerun the model and check for a specific pattern of statistical significance. Therefore, we only considered foraging, nest height and diet as significant if a level was different from all other levels. We plotted all significant variables using the *ggplot2* (Wickham, 2016) package. All values are presented as mean ± s.d., unless otherwise noted.

## Results

### Haemosporidian parasites

From a total of 3569 screened individuals, we found 1469 birds infected with haemosporidian parasites (41% overall prevalence). We found 88 different *Plasmodium* lineages and 64 *Parahaemoproteus* lineages, with *Parahaemoproteus* prevalence marginally higher (16%) than *Plasmodium* (13%).

### Host life-history traits and climatic variables

**Fig. 1.**
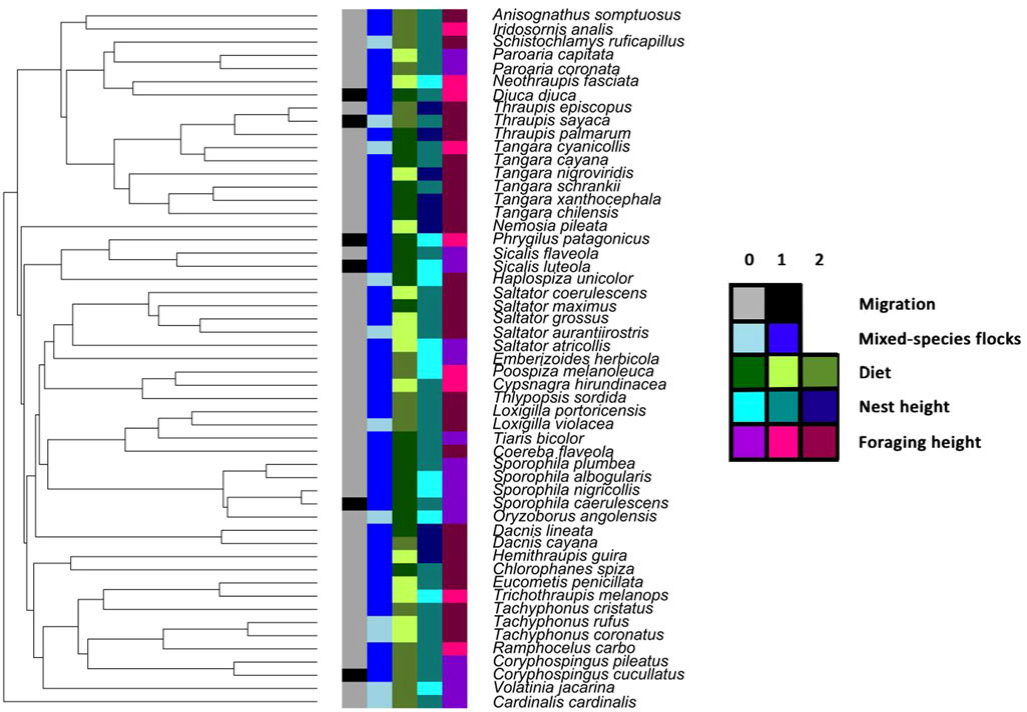
Summary data for categorical life-history variables mapped onto the tips of the trimmed tanager phylogeny, showing as follows: migration (0 – resident; 1 – migrant); mixed-species flocking (0 – non-participant; 1 – participant); diet (0 – plant; 1 – animal; 2 – omnivore); nest height (0 – low; 1 – middle; 2 – high) and foraging height (0 – ground; 1 – understory; 2 – canopy). The colour keys for each category of life-history variables can be seen on the right inset.

**Fig. 2.**
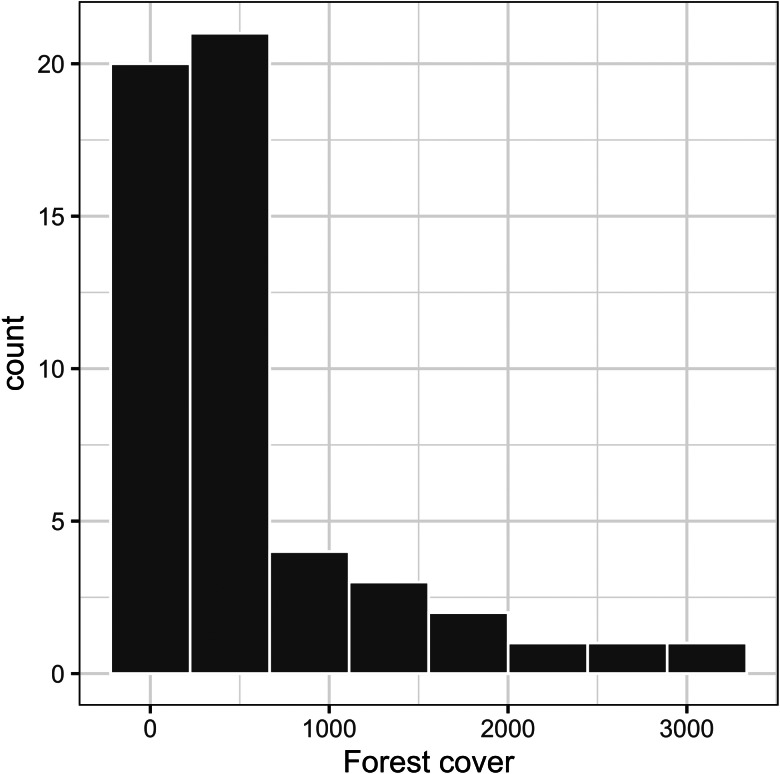
Forest cover histogram multiplied by 0.0001, showing that most species inhabit more open habitats (forest cover closer to zero indicates less forest cover). Forest cover data retrieved from Global Habitat Heterogeneity – dissimilarity index (https://www.earthenv.org/texture), which contains imagery from Moderate Resolution Imaging Spectroradiometer (MODIS) with pixel values collected from satellite images.

We found that most of the tanager species were mixed-species flock participants (79%), non-migratory (88%), middle-forest strata nest builders (60%) and canopy foragers (52%, Fig. 1). Host main diet was well-balanced among the species, with 39% herbivores, 34% omnivores and 27% carnivores (Fig. 1; Supplementary Table 2). Average body size was 14.7 ± 3.2 cm, and incubation period was 13.2 ± 1.0 days. Most of the host species also occurred in more open habitats (Fig. 2).

### Prevalence models

**Fig. 3.**
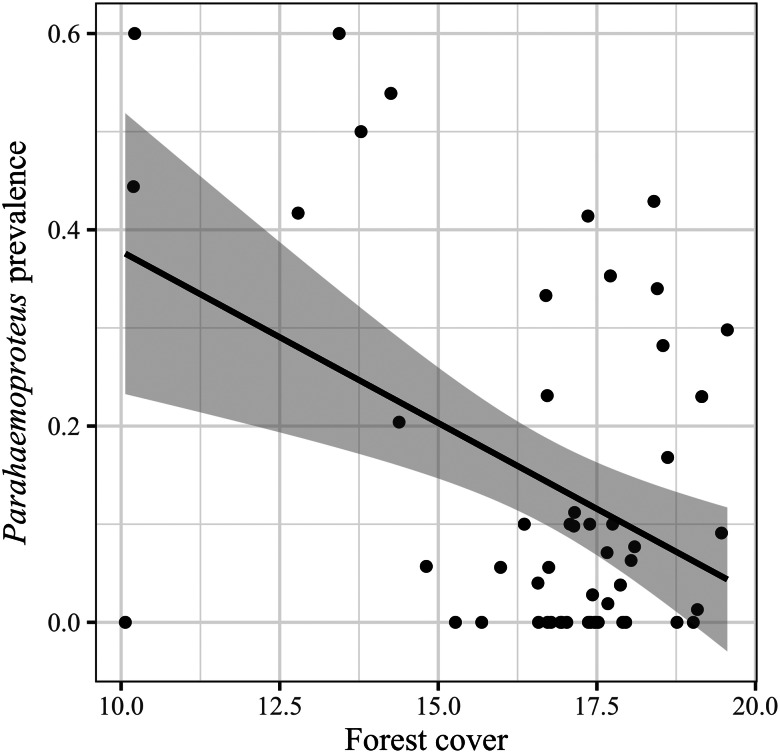
*Parahaemoproteus* prevalence in relation to forest cover (in logarithmic scale) at host specimen collection locations. Points represent the observed values of *Parahaemoproteus* prevalence, and the black line is the fitted curve to the values with the standard error (shaded area).

**Fig. 4.**
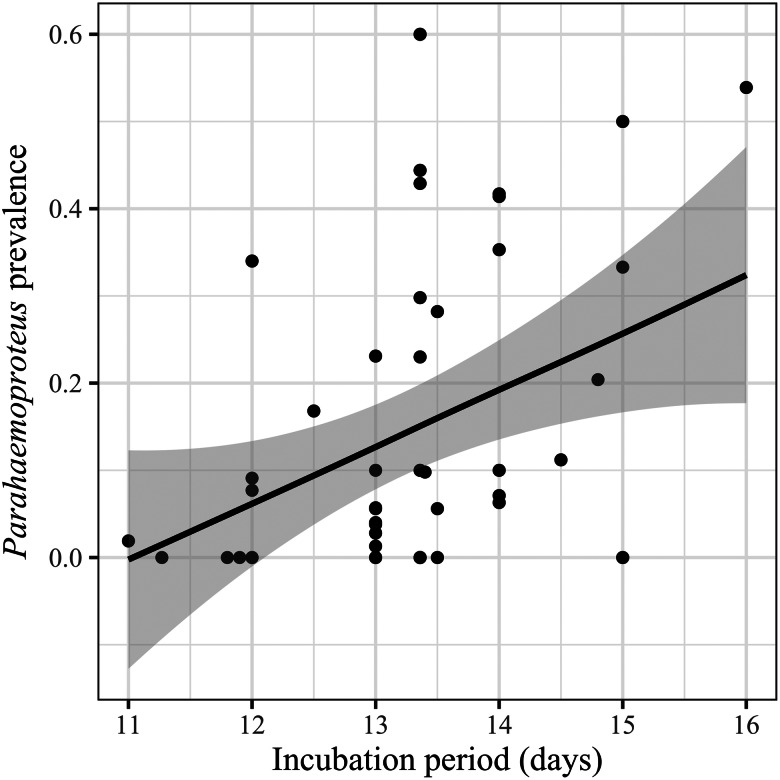
*Parahaemoproteus* prevalence in relation to the incubation period (average number of days). Points represent the observed values for the model incorporating *Parahaemoproteus* prevalence, and the black line is the fitted curve to the values with the standard error (shaded area).

The best models for *Parahaemoproteus* prevalence are presented in Table 1 (RMSE = 0.81). We found higher *Parahaemoproteus* prevalence among tanager species inhabiting areas with less forest cover (Table 2; Fig. 3), and with longer incubation periods (Table 2, Fig. 4). We also found a positive relationship between *Parahaemoproteus* prevalence and incubation period in a more conservative analytical approach (Supplementary Tables 4 and 6).

**Table 1.** Model selection results of *Parahaemoproteus* and *Plasmodium* prevalence (response variables) and the following explanatory variables: climate PC1, climate PC2, body size, mixed-species flock participation, incubation, migration, nest height, foraging height, forest cover and diet

Models	*df*	AICc	ΔAIC	*w_i_*
*Parahaemoproteus*				
Forest cover + incubation	5	−76.7	0.00	0.160
Forest cover + incubation + climate PC1 +climate PC2	7	−74.9	1.77	0.066
Forest cover + migration + incubation	6	−74.9	1.79	0.065
Forest cover + migration + incubation + climate PC1	7	−74.2	2.45	0.047
Forest cover + body size + incubation	6	−74.1	2.59	0.044
Forest cover + mixed-species flock participation + incubation + climate PC1	7	−73.2	3.43	0.029
Forest cover + body size + incubation + climate PC1	7	−72.9	3.76	0.024
Forest cover + migration + body size + incubation	7	−72.9	3.77	0.024
Foraging height + forest cover + body size + incubation	7	−72.8	3.83	0.024
*Plasmodium*				
Diet + forest cover + migration + climate PC1 + climate PC2	9	−93.7	0.00	0.310
Diet + foraging height + forest cover + mixed-species flock participation + incubation + climate PC1 + climate PC2	12	−91.0	2.71	0.080
Diet + forest cover + migration + incubation + climate PC2	9	−90.9	2.79	0.077
Diet + forest cover + migration + incubation + climate PC1 + climate PC2	10	−90.8	2.94	0.071
Diet + foraging height + mixed-species flock participation + body size + climate PC2	10	−90.5	3.19	0.063
Diet + foraging height + migration + climate PC2	9	−90.0	3.76	0.047
Diet + forest cover + migration + mixed-species flock participation + body size + climate PC2	10	−89.9	3.85	0.045

Variables included in each model are shown together with the models' degrees of freedom (*df*), AICc score, delta AIC and weight (*w_i_*). We only show the models with AIC scores lower than 4 for *Parahaemoproteus* and *Plasmodium*. Results for all 53 sampled tanager species in total. Model comparison using OU (*Parahaemoproteus* model AIC = −62.78; *Plasmodium* model AIC = −85.83) and BM (*Parahaemoproteus* model AIC = −35.95; *Plasmodium* model AIC = −56.92), indicated OU as the best in all our models.

**Table 2.** Model-averaged estimates, standard errors and 95% CIs for variables in the model using *Parahaemoproteus* prevalence as the response variable

Variables	Estimate	Standard error	95% CI
Intercept^a^	0.71	0.18	0.34, 1.08*
Forest cover	−0.03	0.00	−0.05, −0.01*
Incubation	0.04	0.01	0.00, 0.07*
Climate PC1	−0.02	0.02	−0.06, 0.01
Climate PC2	−0.00	0.01	−0.03, 0.03
Migration (resident)	0.02	0.03	−0.05, 0.10
Body size	0.00	0.02	−0.05, 0.05
Mixed-species flock participation	0.00	0.03	−0.07, 0.08
Foraging height (ground)	−0.05	0.06	−0.19, 0.07
Foraging height (understory)	0.03	0.05	−0.08, 0.14
Diet (omnivore)	−0.01	0.06	−0.13, 0.11
Diet (plant)	0.01	0.06	−0.11, 0.14
Nest height (low)	−0.04	0.05	−0.14, 0.05
Nest height (middle)	−0.03	0.04	−0.13, 0.05

Significant variables are marked with asterisks. Results for all 53 sampled tanager species in total.

a Reference level for the categorical variables: diet (animal), foraging height (canopy), migration (migrant), nest height (high) and mixed-species flock participation (non-participant).

**Fig. 5.**
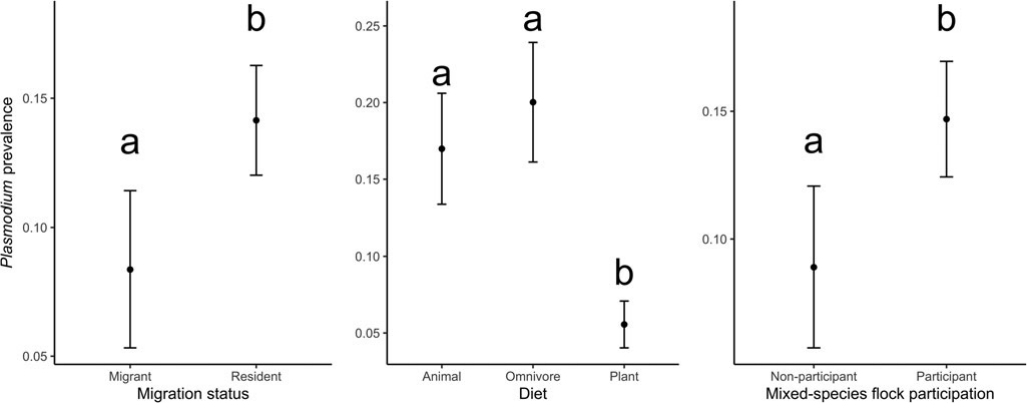
Observed values of *Plasmodium* prevalence in relation to migration status (left) and diet (middle) and mixed-species flock participation (right). Letters indicate statistical difference in prevalence among hosts with different levels of migration status, diet and mixed-species flock participation, meaning that tanager species that migrate, have a plant-derived diet, and do not join mixed-species flocks have lower *Plasmodium* prevalence in comparison with tanager species that are resident, an omnivorous or animal-derived diet, and participate in mixed-species flocks, respectively.

**Fig. 6.**
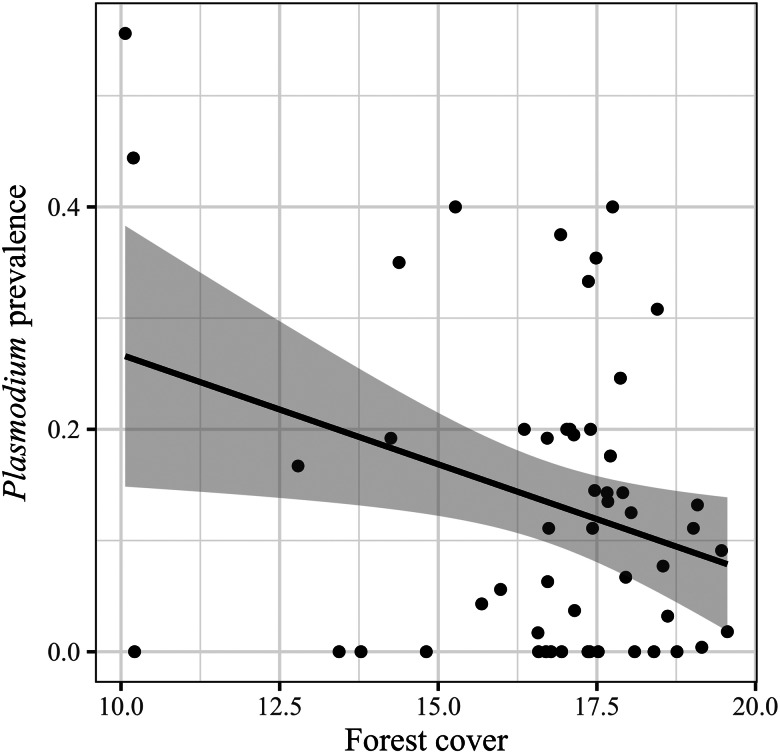
Observed values of *Plasmodium* prevalence in relation to the forest cover. Points represent the values by the model of *Plasmodium* prevalence in relation to forest cover, and the black line is the fitted curve to the values with the standard error (shaded area).

The best models of *Plasmodium* prevalence are shown in Table 1 (RMSE = 0.30). Tanager species without migratory behaviour, with omnivorous or animal-derived diet, mixed-species flocking behaviour (Table 3; Fig. 5) and inhabiting areas with lower forest cover (Table 3; Fig. 6) had higher *Plasmodium* prevalence. We found that mixed-species flock participants had a higher *Plasmodium* prevalence in a more conservative analytical approach (Supplementary Tables 4 and 6).

**Table 3.** Model-averaged estimates, standard errors and 95% CIs of variables in the model using *Plasmodium* prevalence as the response variable

Variables	Estimate	Standard error	95% CI
Intercept^a^	0.39	0.20	0.00, 0.79*
Diet (omnivore)^b^	0.03	0.04	−0.05, 0.11
Diet (plant)^b^	−0.18	0.02	−0.24, −0.12*
Forest cover	−0.01	0.00	−0.02, −0.00*
Migration (resident)	−0.08	0.03	−0.16, −0.01*
Climate PC1	0.01	0.00	−0.00, 0.03
Climate PC2	−0.02	0.01	−0.04, 0.00
Foraging height (ground)^c^	0.06	0.04	−0.01, 0.15
Foraging height (understory)^c^	0.09	0.03	0.02, 0.16*
Mixed-species flock participation	0.08	0.04	0.00, 0.16*
Incubation	−0.01	0.02	−0.05, 0.03
Body size	−0.00	0.01	−0.03, 0.02
Nest height (low)	−0.03	0.07	−0.19, 0.11
Nest height (middle)	0.00	0.04	−0.09, 0.09

Significant variables are marked with an asterisk. Results are for all 53 sampled tanager species in total.

a Reference level for the categorical variables: diet (animal), foraging height (canopy), migration (migrant), nest height (high) and mixed-species flock participation (non-participant).

b Changing the reference level to diet (omnivore): diet (plant): −0.21 ± 0.03 (−0.28, −0.15) and diet (animal): −0.03 ± 0.04 (−0.12, 0.05). Changing the reference level to diet (plant): diet (omnivore): 0.21 ± 0.03 (0.15, 0.28) and diet (animal): 0.18 ± 0.02 (0.13, 0.24).

c Changing the reference level to foraging height (ground): foraging height (understory): 0.01 ± 0.05 (−0.09, 0.11) and foraging height (canopy): −0.08 ± 0.04 (−0.16, 0.00). Changing the reference level to foraging height (understory): foraging height (ground): −0.03 ± 0.05 (−0.14, 0.08) and foraging height (canopy): −0.08 ± 0.04 (−0.18, 0.00).

## Discussion

Overall, we found an association between haemosporidian parasite prevalence and tanagers' life-history traits. Specifically, we found that higher *Parahaemoproteus* prevalence was associated with birds occurring in habitats with lower forest cover (more open habitats), and among birds with longer incubation periods. We also found that *Plasmodium* prevalence was more often associated with birds without migratory behaviour, mixed-species flock participation, with an omnivorous or animal-derived diet and inhabiting less-forested habitats.

We found, first, that *Parahaemoproteus* and *Plasmodium* prevalence was higher in tanager species inhabiting locations with lower forest cover (more open habitats). Habitat type may be an important predictor of haemosporidian parasite prevalence because it may affect the probability of individual birds being exposed to vectors. Haemosporidian parasite vectors are common in nature and have shown some level of host specificity (Martínez-De La Puente *et al*., 2011*a*; Bobeva *et al*., 2015; Tomás *et al*., 2021) and these vectors may change their feeding preferences according to the environmental conditions (Santiago-Alarcon *et al*., 2012). The abundance and prevalence of biting midges can vary with altitude and across and habitat types (open *vs* closed) (Möhlmann *et al*., 2018), which may explain increased probability of infecting tanagers across our sampling locations. Furthermore, vectors of *Plasmodium* have been found to prefer pasture and more open areas in southeastern Brazil (Ferreira *et al*., 2017). However, previous studies have reported contrasting results relating haemosporidian prevalence to habitat type, either showing higher parasite prevalence in open (Reinoso-Pérez *et al*., 2016; Ferreira *et al*., 2017) or in closed habitats (Lutz *et al*., 2015). Our results suggest that tanager species inhabiting places with less forest cover may be more exposed and therefore have an increased likelihood of encountering vectors carrying *Parahaemoproteus* and *Plasmodium* parasites, but future studies should identify these vectors as well as how their differences may vary across habitat types.

Second, we found that tanager species with a longer incubation period had higher *Parahaemoproteus* prevalence; this was the opposite of what we expected. These results were reinforced by findings using more conservative models (*n* > 10 individuals per species) for *Parahaemoproteus* prevalence. A longer incubation period is believed to allow for enhanced development of the immune system (Ricklefs, 1992), with higher B-cell maturation, thus conferring better defence against infections (Ricklefs *et al*., 2018). However, based on our findings we hypothesize that tanager species facing higher selective pressure from *Parahaemoproteus* parasites may trade investing in reproduction over immunity, producing a weaker immune response to fight-off parasites. This is supported by other studies; for example, Palacios and Martin (2006) found that a longer incubation period does not enhance cellular immune response in several passerine bird species. Alternatively, longer incubation periods may increase the chances of attracting vectors of haemosporidian parasites (biting midges for *Parahaemoproteus*; mosquitoes for *Plasmodium*) to incubating adults and their nestlings (Skutch, 1945; Santiago-Alarcon *et al*., 2012) that may lead to more frequent or more efficient parasite infection during this period. Therefore, we also hypothesize that birds with longer incubation periods suffer increased susceptibility to *Parahaemoproteus* vectors among individuals or may attract mosquitoes (*Plasmodium*) more often.

Third, we found that birds joining mixed-species flocks, either frequently or rarely, had higher *Plasmodium* prevalence. Mixed-species flocks are thought to benefit participants through increased foraging success or increased surveillance against potential predators (Zou *et al*., 2018). In the Neotropics, birds often associate with mixed-species flocks after the breeding season to gain potential benefits (Kajiki *et al*., 2018). However, like González *et al*. (2014), we show a positive relationship between flock participation and an increase in the probability of infection by *Plasmodium* parasites. This may be explained by (a) increasing visual or olfactory cues within mixed-species flocks that are in-turn associated with vector attraction (Díez-Fernández *et al*., 2020), or (b) individual birds covering a larger spatial area within flocks resulting in an increased possibility of mosquito encounters (Van Houtan *et al*., 2006).

Fourth, contrary to our original expectations, we found that resident tanager species had a higher *Plasmodium* prevalence compared to migratory tanager species. During migration movements, birds might be more exposed to vectors and, hence, present an increased likelihood of haemosporidian parasite infection (de Angeli Dutra *et al*., 2021). However, non-migrating birds may become more predictably located in space and time, thus increasing their chances of encountering infected vectors year-around. For example, migratory passerines were seldom infected with haemosporidian parasites compared to resident birds in the Dominican Republic (Soares *et al*., 2020). Therefore, our results suggest 2 non-mutually exclusive hypotheses: (a) vectors may have a clear preference and be specialized in resident species, or (b) by encountering sedentary species more often, these species are more likely infected than migratory tanagers. However, because only 12% of our sampled species were migratory (mostly partially migratory), our results should be interpreted with caution; more studies are needed with a larger sample of the family Thraupidae. Furthermore, it is important to emphasize that haemosporidian prevalence, in a particular avian host species, could be an oversimplification as infection probability across individuals' hosts could still depend on other spatial and temporal variables not measured here (e.g. water body availability, bird breeding season), as well as individual development.

Finally, we found that omnivores and tanager species with a more animal-derived diet have a higher *Plasmodium* prevalence compared to those species feeding solely on plant materials. Feeding behaviour is crucial for bird survival, but costs may incur if foraging increases chances of encountering predators (Kelleher *et al*., 2021), or vectors of haemosporidian parasites (Fecchio *et al*., 2022). In fact, our results suggest that birds seeking insects (animal-derived diets and omnivores) may face more encounters with infected vectors with haemosporidian parasites (Ribeiro *et al*., 2005). Furthermore, our results may also indicate that birds with a plant-derived diet have decreased infection chances simply because they have fewer encounters with insects considering that this is not their main feeding resource.

In summary, we found patterns of infection prevalence suggesting that parasitism by haemosporidians is related to a variety of tanager life-history traits. These findings for the host family Thraupidae highlight the difficulty in determining what factors affect parasite prevalence in birds. We suggest 2 non-mutually exclusive approaches to further clarify these relationships and to reveal whether reduced immune response and/or variability in exposure to vectors influences the infection susceptibility of hosts: (1) determining haemosporidian parasite prevalence within relevant vector species in relation to the habitats of avian hosts, and (2) analysing energy trade-offs between immunity and incubation period.

## Data Availability

The authors comply with data availability criterion. Data used in this paper were provided along with R scripts and all datasets used for our analysis.

## Supplementary material

For supplementary material accompanying this paper visit https://doi.org/10.1017/S0031182022001469. click here to view supplementary material
