# Supplementary material for: Host life-history traits predict haemosporidian parasite prevalence in tanagers (Aves: Thraupidae)
Source: Parasitology. 2022 Oct 13;150(1):32–41. doi: 10.1017/S0031182022001469 (PMC10090595; doi:10.1017/S0031182022001469)
Supplement: Supplementary file 1 [file S0031182022001469sup.zip › S0031182022001469sup003.docx]

**Supplementary material**

**Supplementary Table 1:** Species of the Thraupidae sampled including, number of captures, number of infected individuals, prevalence (proportion of infected individuals).

| Species | Captures | Infected | Prevalence |
| --- | --- | --- | --- |
| *Anisognathus somptuosus* | 10 | 6 | 0.60 |
| *Cardinalis cardinalis* | 594 | 394 | 0.66 |
| *Chlorophanes spiza* | 26 | 11 | 0.42 |
| *Coereba flaveola* | 591 | 195 | 0.33 |
| *Coryphospingus cucullatus* | 55 | 18 | 0.32 |
| *Coryphospingus pileatus* | 240 | 136 | 0.56 |
| *Cypsnagra hirundinacea* | 26 | 19 | 0.73 |
| *Dacnis cayana* | 36 | 8 | 0.22 |
| *Dacnis lineata* | 10 | 3 | 0.30 |
| *Diuca diuca* | 39 | 17 | 0.43 |
| *Emberizoides herbicola* | 16 | 1 | 0.06 |
| *Eucometis penicillata* | 5 | 2 | 0.40 |
| *Haplospiza unicolor* | 23 | 1 | 0.04 |
| *Hemithraupis guira* | 10 | 3 | 0.30 |
| *Iridosornis analis* | 9 | 5 | 0.55 |
| *Loxigilla portoricensis* | 29 | 12 | 0.41 |
| *Loxigilla violacea* | 460 | 204 | 0.44 |
| *Nemosia pileata* | 7 | 3 | 0.42 |
| *Neothraupis fasciata* | 137 | 76 | 0.55 |
| *Oryzoborus angolensis* | 28 | 0 | 0.00 |
| *Paroaria capitata* | 76 | 12 | 0.15 |
| *Paroaria coronata* | 18 | 2 | 0.11 |
| *Phrygilus patagonicus* | 57 | 18 | 0.31 |
| *Poospiza melanoleuca* | 7 | 4 | 0.57 |
| *Ramphocelus carbo* | 48 | 18 | 0.37 |
| *Saltator atricollis* | 5 | 2 | 0.40 |
| *Saltator aurantiirostris* | 10 | 1 | 0.10 |
| *Saltator coerulescens* | 16 | 3 | 0.18 |
| *Saltator grossus* | 6 | 0 | 0.00 |
| *Saltator maximus* | 18 | 3 | 0.16 |
| *Schistochlamys ruficapillus* | 36 | 5 | 0.13 |
| *Sicalis flaveola* | 39 | 13 | 0.33 |
| *Sicalis luteola* | 6 | 3 | 0.50 |
| *Sporophila albogularis* | 6 | 2 | 0.33 |
| *Sporophila caerulescens* | 15 | 6 | 0.40 |
| *Sporophila nigricollis* | 6 | 1 | 0.16 |
| *Sporophila plumbea* | 35 | 2 | 0.05 |
| *Tachyphonus coronatus* | 25 | 6 | 0.24 |
| *Tachyphonus cristatus* | 9 | 8 | 0.88 |
| *Tachyphonus rufus* | 215 | 65 | 0.30 |
| *Tangara cayana* | 174 | 34 | 0.19 |
| *Tangara chilensis* | 15 | 5 | 0.33 |
| *Tangara cyanicollis* | 6 | 3 | 0.50 |
| *Tangara nigroviridis* | 12 | 7 | 0.58 |
| *Tangara schrankii* | 17 | 9 | 0.52 |
| *Tangara xanthocephala* | 5 | 3 | 0.60 |
| *Thlypopsis sordida* | 9 | 3 | 0.33 |
| *Thraupis episcopus* | 10 | 5 | 0.50 |
| *Thraupis palmarum* | 14 | 3 | 0.21 |
| *Thraupis sayaca* | 41 | 23 | 0.56 |
| *Tiaris bicolor* | 22 | 4 | 0.18 |
| *Trichothraupis melanops* | 32 | 14 | 0.43 |
| *Volatinia jacarina* | 208 | 68 | 0.32 |

**Supplementary Table 2**: The first and second components (PC1 and PC2) of a principal component analysis result for 19 climate variables.

| Variables | Variable meaning | PC1 | PC2 |
| --- | --- | --- | --- |
| bio1 | Annual mean Temperature | 0.306 | 0.057 |
| bio2 | Mean diurnal range | -0.208 | 0.014 |
| bio3 | Isothermality | 0.161 | 0.011 |
| bio4 | Temperature seasonality | -0.208 | -0.129 |
| bio5 | Maximum Temperature of warmest month | 0.225 | -0.002 |
| bio6 | Minimum Temperature of coldest month | 0.316 | 0.057 |
| bio7 | Temperature annual range | -0.236 | -0.079 |
| bio8 | Mean Temperature of Wettest quarter | 0.267 | 0.038 |
| bio9 | Mean Temperature of driest quarter | 0.314 | 0.075 |
| bio10 | Mean Temperature of warmest quarter | 0.248 | -0.010 |
| bio11 | Mean Temperature of coldest quarter | 0.312 | 0.083 |
| bio12 | Annual precipitation | 0.211 | -0.313 |
| bio13 | Precipitation of wettest month | 0.261 | -0.164 |
| bio14 | Precipitation of driest month | 0.014 | -0.449 |
| bio15 | Precipitation seasonality | 0.156 | 0.330 |
| bio16 | Precipitation of wettest quarter | 0.255 | -0.168 |
| bio17 | Precipitation of driest quarter | 0.021 | -0.454 |
| bio18 | Precipitation of warmest quarter | 0.080 | -0.211 |
| bio19 | Precipitation of coldest quarter | 0.146 | -0.309 |


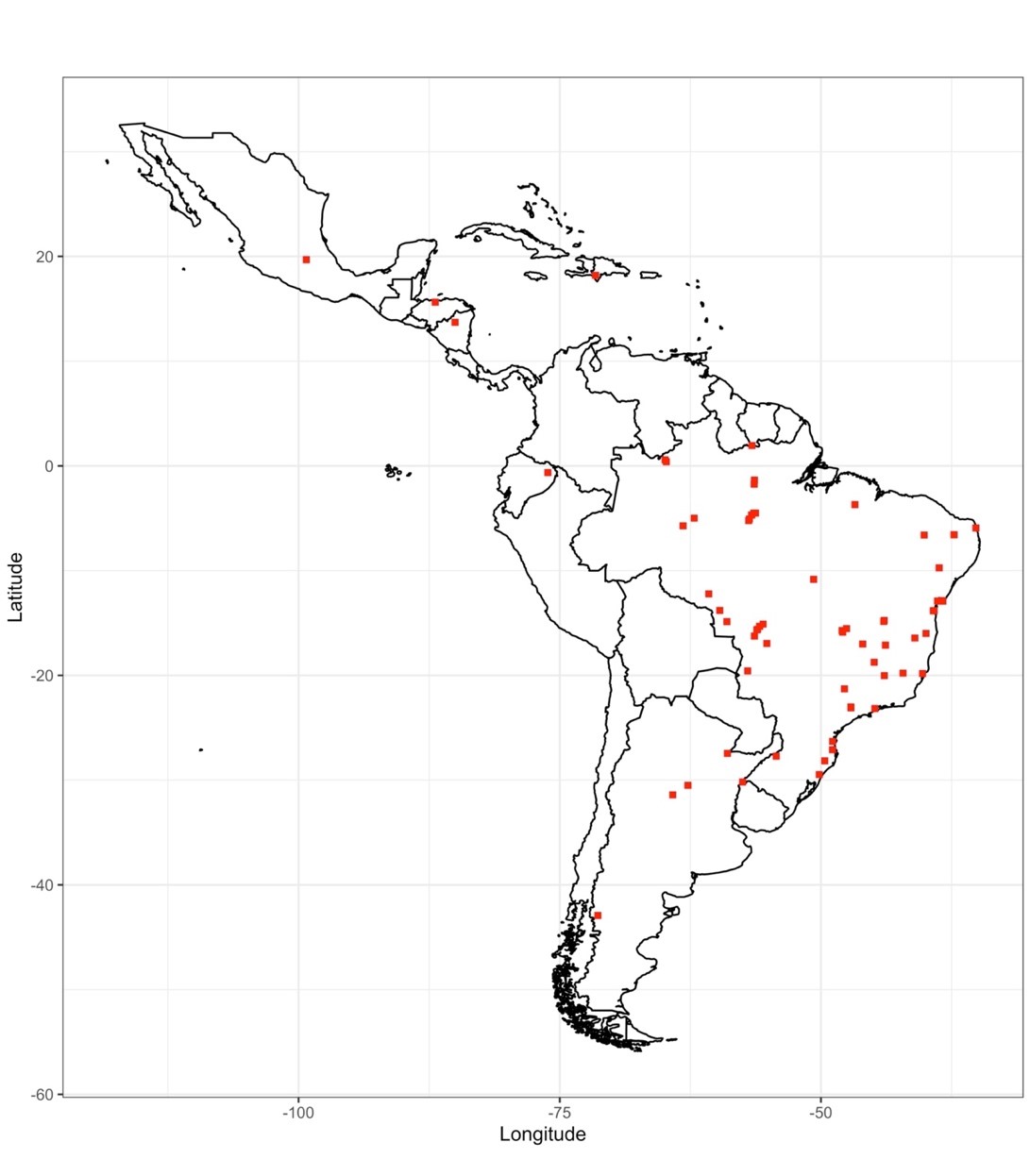


**Supplementary Figure 1:** Sampling locations throughout the Americas, ranging from Mexico to southern South America.


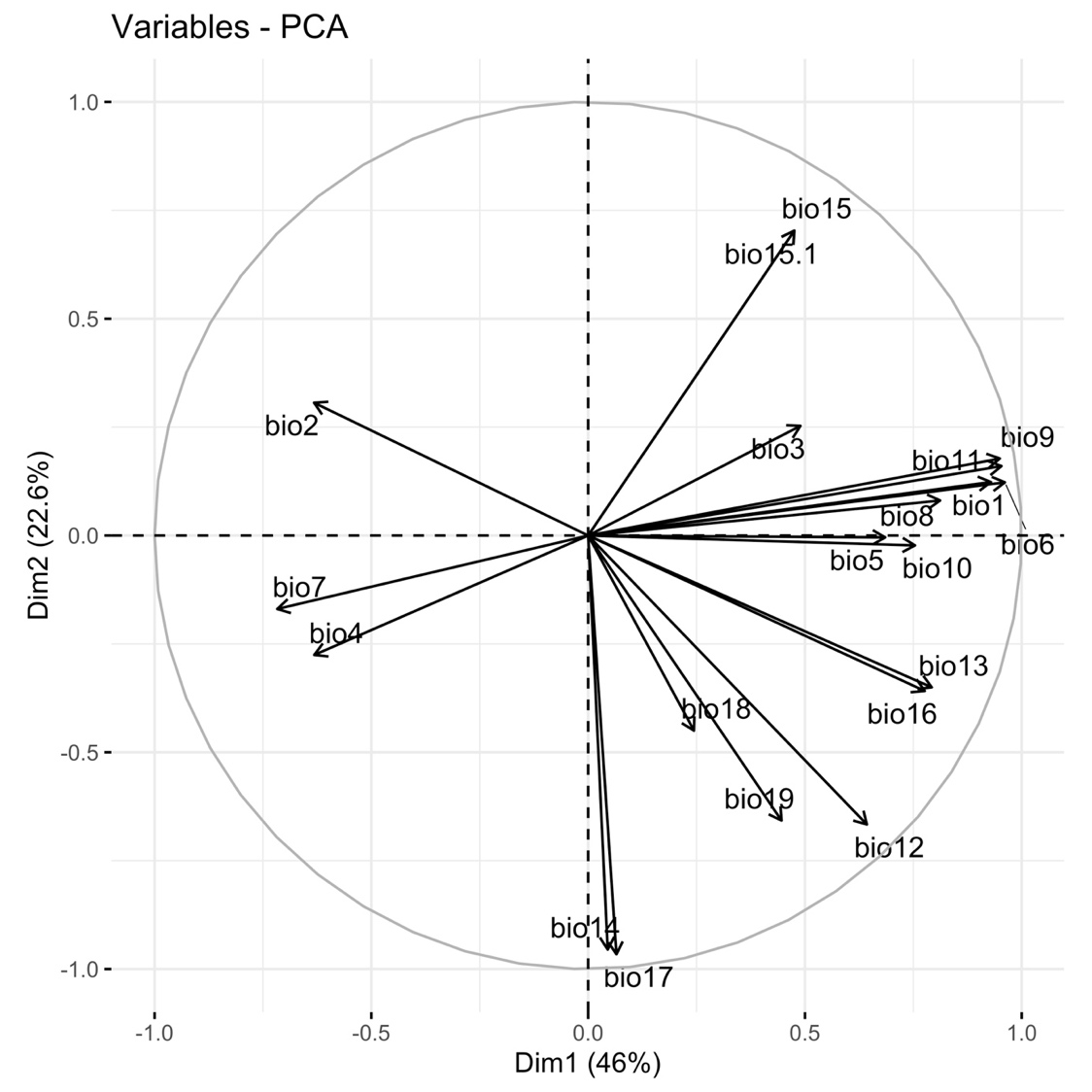


**Supplementary Figure 2**: Biplot of Principal component analysis with all 19 climate variables from WorldClim 2 (Fick and Hijmans, 2017). Here we show the first (x-axis) and second (y-axis) components along with their proportion of explained variability (in parenthesis).

**Supplementary Table 3:** Model selection results of *Parahaemoproteus* prevalence (response variables) and the following explanatory variables: diet (A), foraging height (B), forest cover (C), migratory behavior (D), mixed-species flock participation (E), nest height (F), body size (G), incubation period (H), climate PC1 (I), climate PC2 (J). Variables included in each model are shown together with the models’ degrees of freedom, AICc score, delta AIC, and weight (*wi*). Results for all 40 sampled tanager species with n>10 sampled individuals. We only show models with delta AIC lower than four. Model comparison using OU (-48.11) and BM (-46.92). Symbols (+) indicate that the variable was present in the model.

| A | B | C | D | E | F | G | H | I | J | df | AICc | delta | weight |
| --- | --- | --- | --- | --- | --- | --- | --- | --- | --- | --- | --- | --- | --- |
|  |  |  | + |  |  | + | + | + |  | 6 | -78.074 | 0.000 | 0.209 |
|  |  |  |  | + |  |  | + | + |  | 6 | -77.397 | 0.677 | 0.149 |
|  |  | + |  |  |  |  | + | + |  | 6 | -77.253 | 0.821 | 0.139 |
|  |  | + | + | + |  |  | + | + |  | 8 | -75.869 | 2.206 | 0.069 |
|  |  | + |  |  |  |  | + |  |  | 5 | -75.741 | 2.334 | 0.065 |
|  |  |  | + | + |  |  | + | + |  | 7 | -75.129 | 2.946 | 0.048 |
|  |  | + |  |  |  |  | + | + | + | 7 | -74.242 | 3.832 | 0.031 |
|  |  | + |  |  | + |  | + | + |  | 8 | -74.149 | 3.926 | 0.029 |

**Supplementary Table 4:** Model selection results of *Plasmodium* prevalence (response variables) and the following explanatory variables: diet (A). foraging height (B), forest cover (C), migratory behavior (D), mixed-species flock participation (E), nest height (F), body size (G), incubation period (H), climate PC1 (I), climate PC2 (J). Variables included in each model are shown together with the models’ degrees of freedom, AICc score, delta AIC, and weight (*wi*). Results for all 40 sampled tanager species with n>10 sampled individuals. We only show models with weight delta AIC than four. Model comparison using OU (-48.11) and BM (-46.92). Symbols (+) indicate that the variable was present in the model.

| A | B | C | D | E | F | G | H | I | J | df | AICc | delta | weight |
| --- | --- | --- | --- | --- | --- | --- | --- | --- | --- | --- | --- | --- | --- |
| + |  |  |  | + |  |  |  |  | + | 6 | -56.548 | 0.000 | 0.049 |
| + | + |  | + | + |  |  |  |  | + | 9 | -55.799 | 0.749 | 0.034 |
| + | + |  |  | + |  |  |  |  | + | 8 | -55.149 | 1.399 | 0.024 |
| + |  |  | + | + |  |  |  |  | + | 7 | -55.003 | 1.546 | 0.023 |
| + |  |  |  | + |  |  | + |  | + | 7 | -54.764 | 1.784 | 0.020 |
|  | + |  | + |  |  |  |  |  |  | 5 | -54.494 | 2.054 | 0.018 |
| + |  |  |  | + |  | + |  |  | + | 7 | -54.315 | 2.234 | 0.016 |
|  | + |  | + |  |  |  |  |  | + | 6 | -54.122 | 2.426 | 0.015 |
| + | + |  | + | + |  |  | + |  | + | 10 | -53.922 | 2.626 | 0.013 |
|  | + |  | + | + |  |  |  |  | + | 7 | -53.858 | 2.690 | 0.013 |
| + |  |  |  | + |  |  |  | + | + | 7 | -53.806 | 2.742 | 0.012 |
|  | + |  | + |  |  |  | + |  |  | 6 | -53.802 | 2.747 | 0.012 |
|  | + |  | + | + |  |  |  |  |  | 6 | -53.794 | 2.755 | 0.012 |
| + |  | + |  | + |  |  |  |  | + | 7 | -53.633 | 2.915 | 0.011 |
| + | + |  | + | + |  | + |  |  | + | 10 | -53.618 | 2.930 | 0.011 |
| + |  |  |  | + |  |  |  |  |  | 5 | -53.404 | 3.145 | 0.010 |
| + | + |  |  | + |  |  | + |  | + | 9 | -53.200 | 3.349 | 0.009 |
| + | + |  |  | + |  | + |  |  | + | 9 | -53.178 | 3.370 | 0.009 |
|  | + |  | + | + |  |  | + |  |  | 7 | -53.163 | 3.386 | 0.009 |
| + | + |  | + | + |  |  | + |  |  | 9 | -53.072 | 3.476 | 0.009 |
| + |  |  | + | + |  |  | + |  | + | 8 | -53.031 | 3.518 | 0.008 |
|  |  |  | + | + |  |  |  |  |  | 4 | -53.003 | 3.545 | 0.008 |
| + |  |  | + | + |  |  |  |  |  | 6 | -52.934 | 3.614 | 0.008 |
|  |  |  |  | + |  |  |  |  | + | 4 | -52.792 | 3.756 | 0.007 |
| + | + |  |  | + |  |  |  | + | + | 9 | -52.745 | 3.803 | 0.007 |
| + | + |  | + | + |  |  |  |  |  | 8 | -52.726 | 3.822 | 0.007 |
| + | + |  | + |  |  |  |  |  | + | 8 | -52.668 | 3.880 | 0.007 |
|  | + |  | + |  | + |  |  |  |  | 7 | -52.659 | 3.890 | 0.007 |
|  |  |  |  | + |  |  |  |  |  | 3 | -52.609 | 3.940 | 0.007 |

**Supplementary Table 5**: Model-averaged estimates, standard errors, and 95% confidence intervals for variables in the model using *Parahaemoproteus* prevalence as the response variable. Significant variables are marked with asterisks. Results for all 40 sampled tanager species with n>10 sampled individuals.

| Variables | Estimate | Standard Error | 95% C.I. |
| --- | --- | --- | --- |
| Intercept ^A^ | 0.29 | 0.25 | -0.22, 0.80 |
| Migration (resident) | 0.07 | 0.04 | -0.01, 0.16 |
| Incubation | 0.05 | 0.02 | 0.01, 0.09* |
| Climate PC1 | -0.03 | 0.02 | -0.07, 0.00 |
| Mixed-species flock participation | 0.04 | 0.03 | -0.02, 0.12 |
| Forest cover | -0.02 | 0.01 | -0.04, 0.00 |
| Climate PC2 | -0.01 | 0.02 | -0.05, 0.03 |
| Nest height (low) | 0.04 | 0.09 | -0.14, 0.22 |
| Nest height (middle) | 0.00 | 0.05 | -0.10, 0.11 |
| Diet (omnivore) | 0.07 | 0.05 | -0.03, 0.18 |
| Diet (plant) | 0.00 | 0.05 | -0.10, 0.10 |
| Body size | 0.01 | 0.02 | -0.03, 0.06 |
| Foraging height (ground) | -0.06 | 0.07 | -0.20, 0.08 |
| Foraging height (understory) | 0.05 | 0.07 | -0.08, 0.19 |

^A^ Reference level for the categorical variables: diet (animal), foraging height (canopy), migration (migrant), nest height (high), mixed-species flock participation (non-participant).

**Supplementary Table 6:** Model-averaged estimates, standard errors, and 95% confidence intervals for variables in the model using *Plasmodium* prevalence as the response variable. Significant variables are marked with asterisks. Results for all 40 sampled tanager species with n>10 sampled individuals.

| Variables | Estimate | Standard Error | 95% C.I. |
| --- | --- | --- | --- |
| Intercept ^A^ | 0.06 | 0.13 | -0.21, 0.34 |
| Diet (omnivore) | -0.02 | 0.04 | -0.12, 0.07 |
| Diet (plant) | -0.11 | 0.05 | -0.21, -0.00* |
| Mixed-species flock participation | 0.08 | 0.04 | 0.00, 0.17* |
| Climate PC2 | -0.03 | 0.01 | -0.07, 0.00 |
| Foraging height (ground) | 0.03 | 0.05 | -0.08, 0.15 |
| Foraging height (understory) | 0.15 | 0.06 | 0.02, 0.28* |
| Migration (resident) | 0.07 | 0.04 | -0.01, 0.16 |
| Incubation | -0.02 | 0.02 | -0.07, 0.02 |
| Body size | -0.00 | 0.02 | -0.06, 0.04 |
| Climate PC1 | 0.01 | 0.02 | -0.02, 0.05 |
| Forest cover | 0.00 | 0.01 | -0.02, 0.03 |
| Nest height (low) | -0.08 | 0.08 | -0.25, 0.09 |
| Nest height (middle) | -0.07 | 0.05 | -0.18, 0.03 |

^A^ Reference level for the categorical variables: diet (animal), foraging height (canopy), migration (migrant), nest height (high), mixed-species flock participation (non-participant).

^B^ Changing the reference level to diet (omnivore): diet (plant): -0.08 + 0.04 (-0.17, 0.00), diet (animal): 0.02 + 0.04 (-0.07, 0.12). Changing the reference level to diet (plant): diet (omnivore): 0.08 + 0.04 (-0.00, 0.17), diet (animal): 0.11 + 0.05 (0.00, 0.21).

^C^ Changing the reference level to foraging height (ground): foraging height (understory): 0.12 + 0.07 (-0.02, 0.26), foraging height (canopy): -0.03 + 0.05 (-0.15, 0.08). Changing the reference level to foraging height (understory): foraging height (ground): -0.12 + 0.07 (-0.26, 0.02), foraging height (canopy): -0.15 + 0.06 (-0.28, -0.02).
